# No effect of subthalamic deep brain stimulation on metacognition in Parkinson’s disease

**DOI:** 10.1038/s41598-022-26980-8

**Published:** 2023-01-02

**Authors:** Carlos Trenado, Matthias Boschheidgen, Karim N’Diaye, Alfons Schnitzler, Luc Mallet, Lars Wojtecki

**Affiliations:** 1 Institute of Clinical Neuroscience and Medical Psychology, Medical Faculty, Heinrich Heine University, Duesseldorf, Germany; 2 Max Planck Institute for Empirical Aesthetics, Frankfurt am Main, Germany; 3 Departmemt of Neurology and Neurorehabilitation, Hospital Zum Heiligen Geist, Academic Teaching Hospital of the Heinrich-Heine-University Duesseldorf, von-Broichhausen-Allee 1, 47906 Kempen, Germany; 4 Institut du Cerveau et de la Moelle Épinière, Hôpital Pitié-Salpêtrière, Paris, France; 5 Department of Mental Health and Psychiatry, Global Health Institute, University of Geneva, Geneva, Switzerland

**Keywords:** Neuroscience, Cognitive neuroscience, Movement disorders, Parkinson's disease

## Abstract

Deep brain stimulation of the subthalamic nucleus (STN-DBS) is a powerful treatment in Parkinson’s disease (PD), which provides a positive effect on motor symptoms although the way it operates on high cognitive processes such as metacognition remains unclear. To address this issue, we recorded electroencephalogram (EEG) of PD patients treated with STN-DBS that performed a reversal learning (RL) paradigm endowed with metacognitive self-assessment. We considered two stimulation conditions, namely DBS-ON (stimulation on) and DBS-OFF (stimulation off), and focused our EEG-analysis on the frontal brain region due to its involvement on high cognitive processes. We found a trend towards a significant difference in RL ability between stimulation conditions. STN-DBS showed no effect on metacognition, although a significant association between accuracy and decision confidence level held for DBS OFF, but not in the case of DBS ON. In summary, our study revealed no significant effect of STN-DBS on RL or metacognition.

## Introduction

Deep Brain Stimulation (DBS) of the subthalamic nucleus (STN) is a technique for the treatment of neurological and psychiatric disorders such as Parkinson’s disease (PD)^1,2^ or obsessive compulsive disorder (OCD)^3^. Although the effect of DBS on motor symptoms such as bradykinesia, muscle rigor or resting tremor has been extensively addressed^4^, its side effects on cognition remain a field of ongoing research. In general, DBS has been assumed to modulate cortico-striatal circuitries with direct effect on motor functions and indirect effect in cognitive and behavioral abilities^5,6^.

Reversal learning (RL) tasks have been extensively used to investigate behavioral flexibility and set-shifting in PD patients under levodopa and stimulation therapy^7–12^. RL requires participants to discriminate between two different stimuli with fixed reward contingencies, acquire and apply a strategy. After reaching a specific reversal criterion, contingencies change and subjects have to adapt their strategy and learn the new rule. In particular, an impairment of RL in PD patients has been previously reported^10^. With regard to the effect of levodopa, several studies showed an impairment of RL and enhanced reward-seeking-behavior^8,11,13^. Others investigated RL in PD patients with bilateral STN-DBS three months after surgery and found an improvement of performance in a reversal/extinction task^9^. Focusing on PD’s neural mechanism, it has been suggested that basal ganglia-thalamo-cortical-circuits play an important role not only in motor, but also in cognitive and behavioral functions. Specially frontostriatal and orbitofrontal loops seem to be involved in decision-making processes and thus have an influence on performance in reversal learning^14,15^. In relation to this, Cavanagh et al. showed a speeding up of decision making in high conflict situations^16^ and suggested that the STN works as a control of cortico-striatal function, thus enabling accumulation of evidence that leads to a well-considered decision. By focusing on the effect of STN-DBS, it has been reported that PD patients tend to speed up their decision-making thus leading to impulsivity symptoms. On the other hand, some studies reported an improvement in decision-making under risky situations^17^.

Metacognition is the process of examining one’s own thoughts and opinions which plays a crucial role in learning and decision making^18^. Previous studies have emphasized the importance of self-awareness and metacognitive skills in PD patients^19–22^. For instance, a comparative study in PD patients with and without impulse control disorders (ICD) reported comparable or increased level of self-awareness in the ICD group. The awareness of the inability to resists impulse behaviors in the ICD group was hypothesized as a source of increased depression in PD patients with ICD^20^. Oh-Lee et al. reported that memory and metacognitive function are not affected in PD patients in a Tip-of-Tongue experiment^21^.

In a previous publication^18^, we already utilized a RL paradigm endowed with metacognitive self-assessment to assess behavioral flexibility and metacognition in a group of PD patients and found a non-compromising positive effect of dopaminergic medication on metacognition. Now we present data of a PD cohort undergoing STN-DBS while being assessed with a similar paradigm. Since the STN receives inputs from frontal cortical areas (via the hyperdirect pathway) and such regions have been implicated in behavioral flexibility and metacognition, it is relevant to gain understanding on the prospective effect of STN-DBS on these cognitive processes and its neurophysiological manifestation. We expected speeding up of decision-making in trials with low confidence rating due to STN-DBS with an effect on metacognitive processes.

## Methods

### Patients

Fourteen PD patients with at least three months since the time of STN-DBS implantation were recruited from the movement disorders unit of the University Hospital Düsseldorf, although only twelve patients (2 female and 10 male) were able to complete the study. The sample size was based on our previous metacognition study in PD^18^ that showed effects on a reversal learning task by including 10 PD patients. All Patients had no previous experience with a RL task. Dementia and major depression were ruled out based on psychological assessment (all patients had a score > 132 on the Mattis Dementia Rating Scale (MDRS) and < 18 on the Beck Depression Inventory (BDI)^23,24^. Furthermore, patients did not suffer from any other psychological disease or movement disorder. Table 1 provides patient’s demographic information, stimulation settings, disease duration, psychological scores. The present study was in agreement with the Helsinki Declaration and approved by the ethics committee of the University Hospital Düsseldorf (Study Nr. 3209). Patients that participated in the study signed an informed consent form.

**Table 1:** Patient information: sex, mean score of the Beck depression inventory (BDI), mean score of the positive and negative affect schedule (PANAS), mean score of the Mattis dementia rating scale (MDRS), UPDRS scores under effect of stimulation (DBS-ON) and no stimulation (DBS-OFF), disease duration with respect to the date of operation (years).

Patient	Sex	BDI	PANAS (+)	PANAS (−)	MDRS	UPDRS DBS-ON	UPDRS DBS-OFF	Disease duration
PAT_1	m	4	35	10	138	38	46	15
PAT_2	m	6	28	11	142	13	30	12
PAT_3	w	9	26	11	142	5	25	12
PAT_4	w	7	45	11	143	15	24	6
PAT_5	m	1	31	11	143	3	10	6
PAT_6	m	14	27	11	142	22	34	6
PAT_7	m	6	31	11	144	6	19	3
PAT_8	m	6	33	10	142	14	38	8
PAT_9	m	10	35	20	136	5	11	9
PAT_10	m	8	28	14	136	17	58	16
PAT_11	m	7	29	14	136	15	26	6
PAT_12	m	6	30	15	133	37	48	13

### Study design

The day before the test, patients received information about the study, signed an informed consent form for participation and performed the neuropsychological tests. On the same day, patients suspended their daily anti-parkinsonian medication. The next day, participants received instructions about how to perform the RL task. Subsequently, we performed EEG preparation steps, including cap appropriate placement and control of electrode impedances. With regard to DBS, two experimental conditions were considered in randomized order: DBS-OFF (stimulation off) and DBS-ON (stimulation on). Note that both experimental conditions were performed off medication. The experiment lasted approximately 30 min depending on the performance of participants. Patients that started the experiment with the DBS-ON condition took a break of at least 30 min since the switching off stimulator before the start of the next experimental session to exclude any stimulation effects.

### Neuropsychological screening

We assessed depression symptoms with the Beck Depression Inventory (score range: 0–63). A cut-off score above 18 was adopted for clinically relevant depression. We assessed positive (PA) and negative affect (NA) with the PANAS questionnaire^25^ (score ranges for PA and NA: 10–50). Assessment of Dementia was done with the Mattis Dementia Rating Scale (MDRS) (maximum score: 144) with a cut-off-score of 132/144. Table 1 displays neuropsychological scores of patients.

### Motor tests

MDS-UPDRS scores were obtained for each patient after each testing condition (See Table 1).

### Reversal leaning task

**Figure 1:**
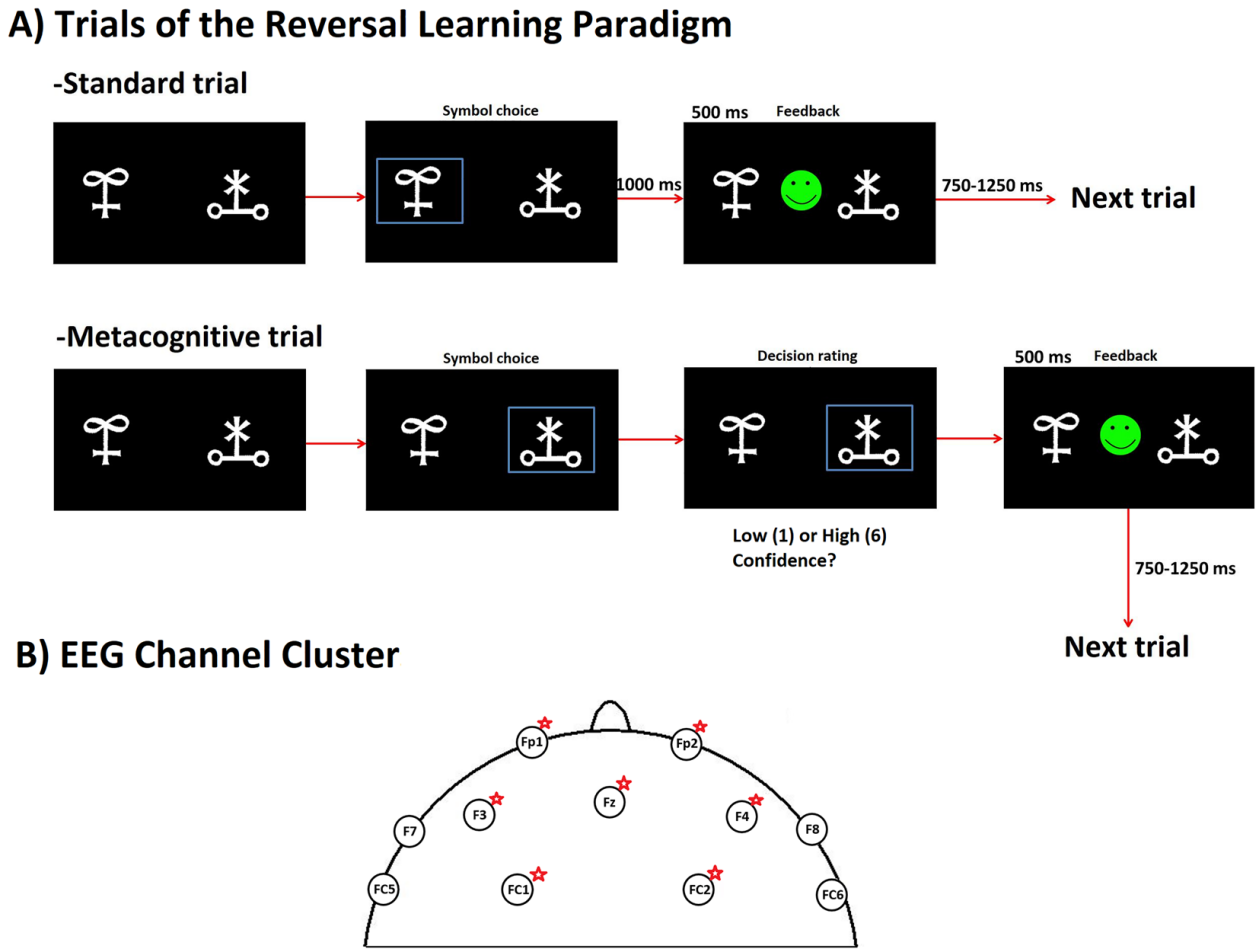
Reversal Learning (RL) Paradigm and EEG Clusters. (**A**) The RL task consisted of trials with and without metacognitive assessment, which we termed as Metacognitive and Standard trials, respectively. The order of trial presentation was randomized. (**B**) Two EEG clusters consisting of channels (FP1, FP2, F3, Fz, F4, Fc1, Fc2) and (F3, Fz, F4) were considered for further analysis.

The RL task utilized in the present study was implemented by using the Psychotoolbox software (http://psychtoolbox.org/). Stimuli consisted of pairs of Hiragana characters (Fig. 1 A) displayed on a 19″ computer monitor placed 50 cm apart from participants. In each trial, participants selected one of two symbols displayed in the screen by pressing a key on a keyboard. Stimuli remained on the screen until subjects responded. After the response, a happy or sad smiley was displayed for 500 ms to provide participants with feedback. At random trials, participants were asked if they were confident about their response (low confidence corresponds to level 1 and high confidence corresponds to level 6). The inter-trial interval was set up to 500 ms. Contingencies between two symbols were fixed to a rate 80/20, namely the rewarded symbol was followed by positive feedback except in 20% of the trials. Probabilistic error was defined for trials when subjects chose the rewarded symbol and subsequently received negative feedback. The criterion for reversal was as follows: after completion of 8 right out of 10 trials, a reversal was set up to occur with probability 25% for each new trial until reaching the maximum number of 15 right trials. As we used a probabilistic RL task, a correct symbol was not always followed by positive feedback. If participants chose the wrong symbol before reaching the specified number of consecutive right trials, they had to begin again. Participants were asked to change their decision only when they were completely sure that contingencies had changed, taking into consideration that probabilistic error could occur.

Each participant completed between 20 and 30 blocks. Each block consisted of between 11 and 100 trials depending on the patient’s interest on the task and clinical state. There was a break after block 10 and 20. After each break, the Hiragana characters in use were replaced by new ones. Probabilistic error could not occur in the first three trials after reversal. Three or less consecutive probabilistic errors were possible during blocks so patients could not follow a specific strategy (e.g. change after second negative feedback).

### EEG recording

Electroencephalogram (EEG) (bandpass 0.5-1000 Hz, sampling rate 5000 Hz) was recorded (BrainVision, BrainAmp MR plus, Brain Products GmbH, Munich, Germany) from 32 electrode positions (BrainCap MR, Brain Products GmbH, Munich, Germany) referenced to FCz and with ground at AFz according to the 10–20 system^26^. Whenever possible, electrode impedances were kept below 5Kohm. EEG events were defined by markers automatically generated by the RL task. In particular, we focused on trials between stimulus presentation and symbol selection, which is the period in which patients experienced more uncertainty in their decision.

### Data analysis

#### Behavioral data

With regard to behavior, we considered the following RL parameters: (1) number of trials to reach reversal (TR); (2) number of consecutive errors after reversal (CEAR); (3) percentage of random switch (RS) (trials were patients switched symbols after getting positive feedback); (4) strategy change after probabilistic error (SCAPE) (trials after probabilistic error were patients switched symbols directly). With regard to metacognition, we considered: (5) decision confidence level. With regard to performance, we considered: (6) response time (RT) and (7) accuracy.

#### Metacognitive data

We focused on the relationship between confidence rating scale and accuracy by statistical tests (Chi-square test). Moreover, metacognition type 2 sensitivity (meta-d), i.e. the efficacy with which observers’ confidence ratings discriminated between their own correct and incorrect stimulus classification, was assessed by type 2 signal detection theory (SDT)^27^.

#### Statistical analysis

Focusing on RL data, we employed non-parametric tests as our data showed significant deviation from normality as indicated by the Shapiro–Wilk test. In particular, Mann–Whitney U test was utilized for comparisons between independent groups, while comparison between dependent groups was performed by the Wilcoxon signed-rank test.

Effect of stimulation on different RL parameters was addressed by the Friedman test. In order to determine a relationship between categorical variables (confidence rating and accuracy), we made use of the Chi-square test.

Focusing on electrophysiological data, we used repeated measures ANOVA with factors stimulation and confidence level. We performed post hoc tests for dependent and independent sample comparisons. Furthermore, assessment of possible correlates of uncertainty was achieved through one-way ANOVA with the factor security level.

Statistical analyses were performed by using the software IBM SPSS Statistics (Version 24, IBM Software, Business and analytics, Armonk, NY, USA). The significance level for all statistical tests was set up at 0.05.

#### EEG analysis

EEG data was band pass filtered (0.3-70 Hz) and subsequently notch-filtered (50 Hz) to remove line disturbances. Next, we conducted a manual raw data inspection to eliminate technical artefacts. We eliminated muscle and eye activity as well as DBS disturbances by means of independent component analysis (ICA). A maximum of four ICA components were removed to keep as possible the integrity of the data. Finally, we visually inspected the data to remove any remaining disturbances. Data was segmented based on markers generated by the RL paradigm. We particularly focussed on trials were self-assessment was collected and segments between stimulus presentation and selection of symbol, as decision-making process occurred in this period and uncertainty could be monitored in relation to an electrophysiological correlate. Afterwards, we performed Fast Fourier Transformation (FFT) and power analysis of the EEG signals. Power analysis was extracted from a frontal symmetrical cluster consisting of seven scalp channels (FP1, FP2, F3, Fz, F4, FC1, FC2) (Fig. 1B). Area under the curve (AUC) corresponding to the power curve was calculated for different frequency bands [Delta (1-3 Hz), Theta (4-7 Hz), Alpha (8-12 Hz), Beta (13-30 Hz)]. Z-standardisation was performed to avoid differences due to stimulation effects. Analysis of electrophysiological data was conducted with *Brain Vision Analyzer* (BrainProducts GmbH, München).

## Results

### Reversal learning parameters

**Figure 2:**
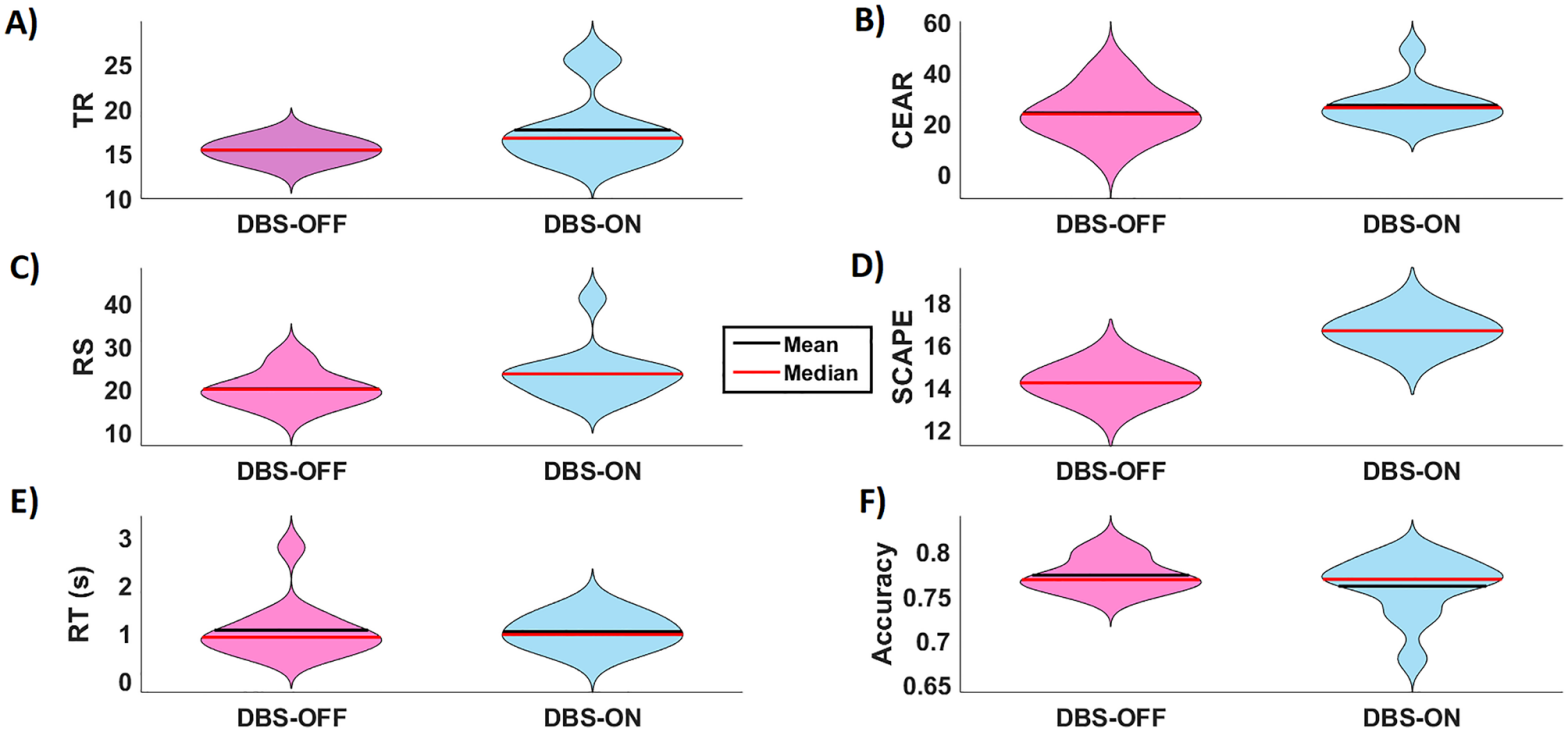
Effect of DBS on reversal Learning (RL) and performance parameters. The violin- type graphs depict the distribution, mean and median of each variable for both stimulation conditions (DBS-OFF and DBS-ON). (**A**) number of trials to reach reversal (TR); (**B**) number of consecutive errors after reversal (CEAR); (**C**) number of random switch (RS) (trials were patients switched symbols after getting positive feedback); (**D**) strategy change after probabilistic error (SCAPE) (trials after probabilistic error were patients switched symbols directly); (**E**) response time (RT) and (**F**) accuracy.

We found a trend towards significant difference in number of trials to reach reversal (TR) between DBS-OFF (15.39 ± 1.12) and DBS-ON (17.62 ± 3.93) as revealed by Wilcoxon-signed-rank-test (Z =  − 1.647; *p* = 0.099) (Fig. 2A). We found a trend towards significant difference in random switch between DBS-OFF (20.08 ± 3.92) and DBS-ON (23.50 ± 6.26) as revealed by Wilcoxon-signed-rank-test (Z =  − 1.785; *p* = 0.074) (Fig. 2C). No significant differences were revealed for other RL parameters (Fig. 2B and D). STN-DBS had no significant effect on RL parameters.

### Performance

We found no significant difference in task performance parameters, e.g. reaction time (DBS-OFF 1.06 ± 0.610152604; DBS-ON1.03 ± 0.311) (Fig. 2E) and accuracy (DBS-OFF 0.77 ± 0.019; DBS-ON 0.76 ± 0.034) (Fig. 2F) between DBS-ON and DBS-OFF.

### Metacognition

Pearson chi-square test revealed a significant relationship between confidence level and accuracy for the DBS-OFF group (*p* < 0.05), which was not held in the case of the DBS-ON group (*p* > 0.733).

We utilized signal detection theory type 2 to assess metacognitive performance. As such, we calculated metacognition type 2 sensitivity (meta-d) and stimulus discrimination sensitivity (d) for each participant. We found a trend towards positive correlation between d and meta-d in the case of DBS-OFF (r = 0.497, *p* = 0.10) which did not follow in the case of DBS-ON group (r =  − 0.114, *p* = 0.725). In relation to this, we found a significant association between accuracy and decision confidence level as revealed by the chi-square test for DBS-OFF (*p* = 0.04). Likewise, this association was not present in DBS-ON.

**Figure 3:**
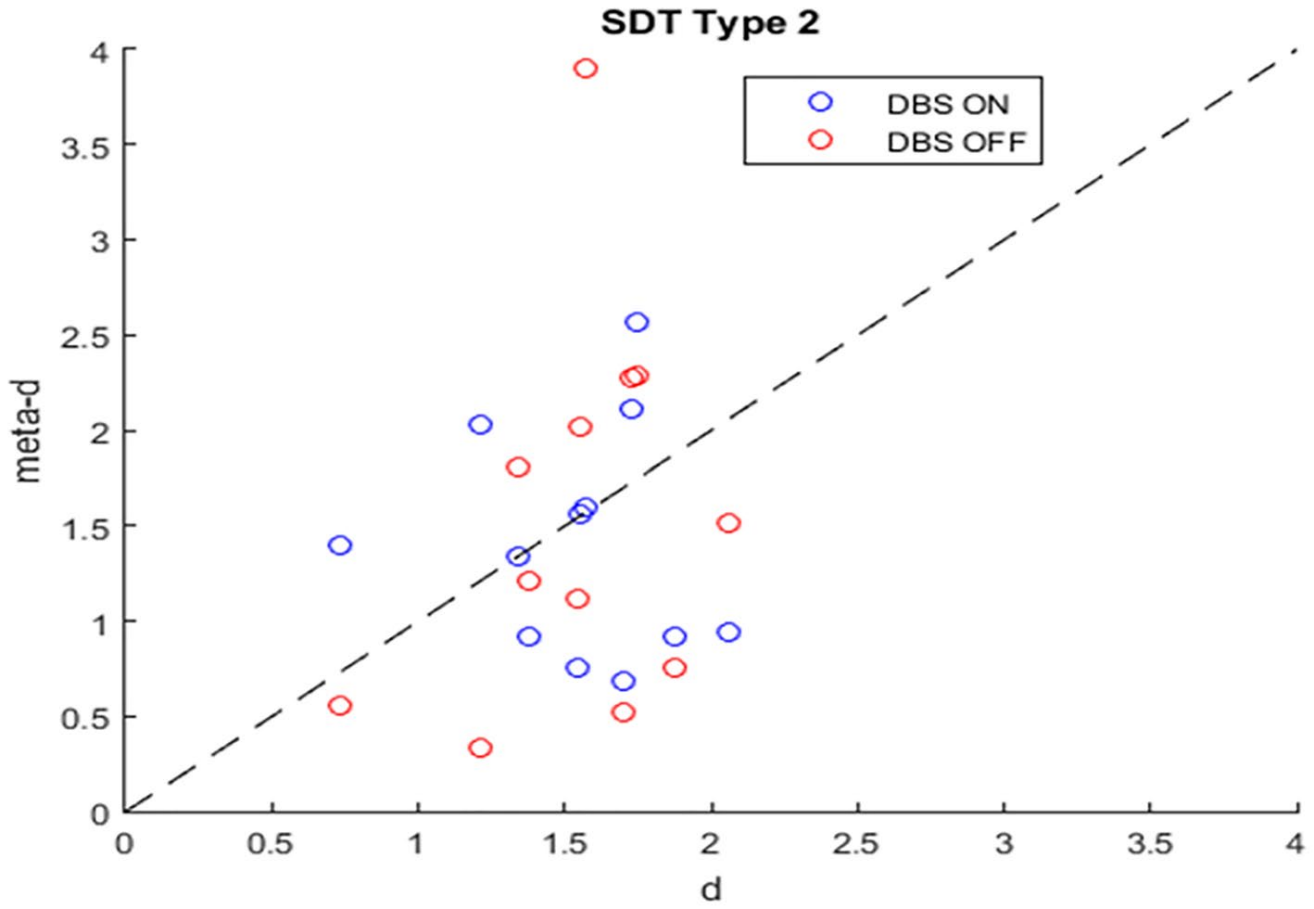
Analysis of relative type 2 sensitivity: meta-d (observed type 2 sensitivity) and d (type 1 sensitivity) for each subject across conditions (DBS-OFF and DBS-ON). We computed such parameters by using the methodology provided by Maniscalco and Lau (2012). Note that if meta-d = d, then a subject is “ideal” from a metacognitive perspective, while the degree to which meta-d is smaller than d reflects the degree to which the subject is inefficient in metacognitive terms.

Seven patients outperformed or were in agreement with SDT expectation (meta-d >  = d) in the DBS-ON group and five patients in the DBS-OFF group (Fig. 3). The mean level of d was 1.664, the mean level of meta-d was 1.524 for DBS-ON. The mean level of d was 1.537, the mean level of meta-d was 1.402 for DBS-OFF. A paired t-test revealed no significant difference between the mean level of meta-d and the mean d for DBS-ON or DBS-OFF.

The mean values of meta-d/d were 0.8936 (Stim-OFF) and 0.9837 (Stim-ON). This means that the average patients exhibited absolute type 2 sensitivity at 89.36% of what would have been expected from their type 1 task performance in DBS-OFF. Deep Brain Stimulation improved type 2 sensitivity to a 98.37% of the estimated type 1 performance. The difference was not significant.

### Electrophysiological data

#### Effect of stimulation

**Figure 4:**
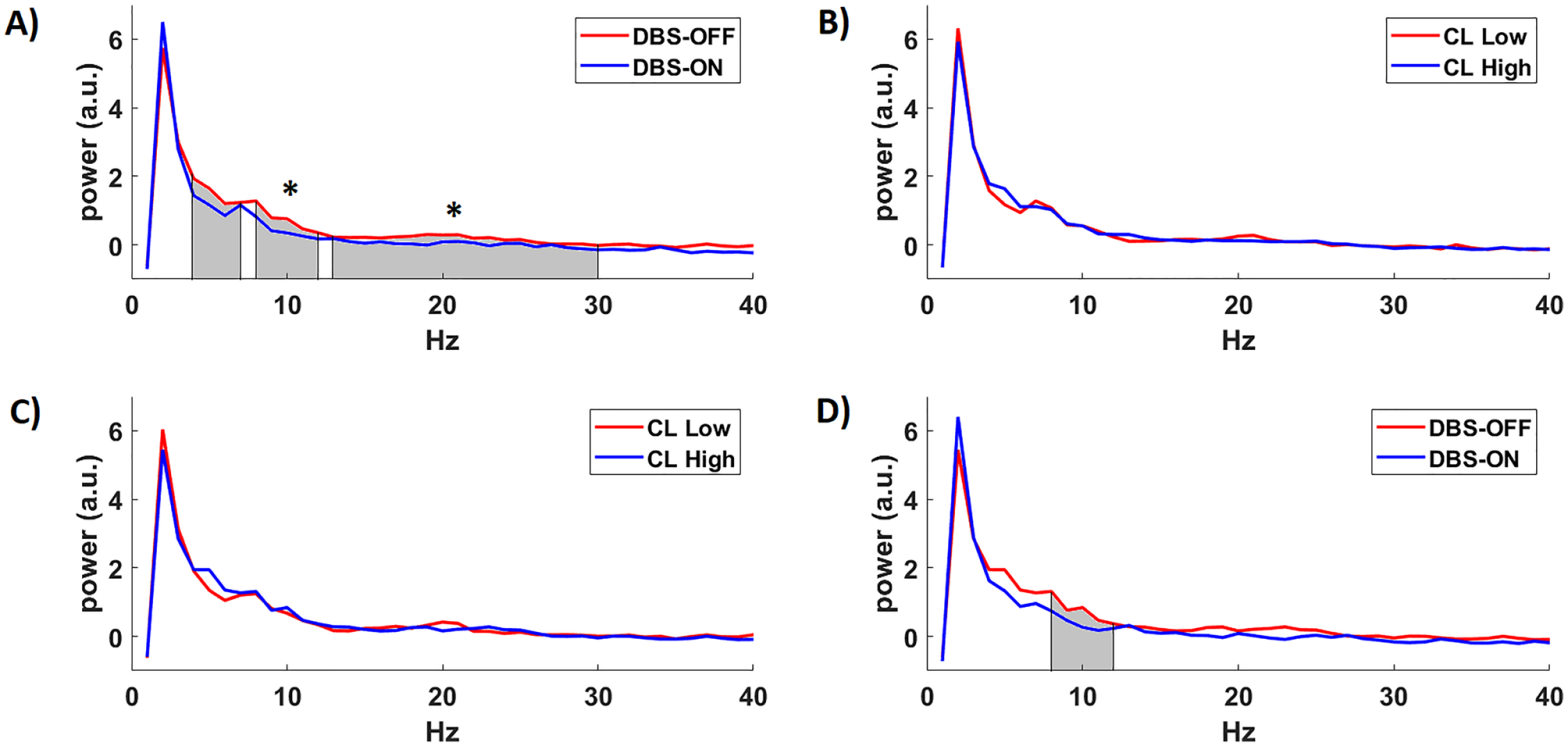
Electrophysiological results. The graphs depict mean power across patients by comparing power of trials corresponding to (**A**) DBS-OFF and DBS-ON regardless of the confidence level to identify a marker of stimulation, (**B**) low and high confidence level regardless of stimulation condition to identify a marker of confidence; (**C**) low and high confidence level under no effect of stimulation and (**D**) DBS-ON and DBS-OFF by considering a high level of confidence. For the calculation of power, we considered a 7-EEG channel frontal cluster.

Repeated measures ANOVA revealed a significant within-subjects effect for the factor stimulation (*p* < 0.05), but no for the factor confidence level (*p* > 0.05). Figure 4A depicts a significant difference in power between DBS-OFF and DBS ON regardless of the confidence level for α- (F = 7.208, *p* = 0.011) and β-band (F = 6.615, *p* = 0.014) as well as a trend for θ-band (F = 3.336, *p* = 0.076) by using the 7-channel frontal cluster.

#### Effect on confidence level (CL)

We performed a comparison in power between trials corresponding to high and low confidence level regardless of stimulation to identify an electrophysiological marker related to confidence. We found no significant difference in power between confidence levels by using the 7-channel frontal cluster (Fig. 4B).

In order to test a prospective relationship in power between the levels of confidence under no effect of stimulation, we focused on trials in which patients assessed their choice under DBS-OFF. We found no significant difference in confidence by using the 7-channel frontal cluster (Fig. 4C). Likewise, we found no significant differences for the DBS-ON condition.

#### Comparison between stimulation conditions on trials with high confidence

We compared power between conditions DBS ON and DBS OFF by considering trials with high level of confidence. One-way ANOVA revealed a trend toward significant difference in power between conditions for the α-band (F = 4.153, *p* = 0.054) (see Fig. 4D).

## Discussion

To our knowledge, this is the first study that examined reversal learning with endowed metacognitive self-assessment in a group of PD patients treated with STN-DBS. In particular, we assessed metacognitive ability in PD patients, the prospective effect of DBS on such high cognitive function and its neurophysiological manifestation as reflected in electroencephalographic recordings.

In agreement with previous RL studies in rats, we showed a trend towards an impairment in behavioral flexibility due to DBS^28^. Note that Funkiewicz et al. used a reversal/extinction-task in PD patients treated with DBS and found a significant positive effect of stimulation in the extinction-phase of the experiment^9^. Regarding the effect of STN-DBS on decision making, Frank et al. hypothesized that the STN functions as a stop mechanism in the decision process, which enables reaching an appropriate decision by buying precious time^15,29^. Consequently, it has been suggested that DBS impairs this important function by leading to a more impulsive behavior in PD patients as similarly shown by other studies^16,30^. In agreement with this, we found a significant association between accuracy and confidence level for DBS-OFF which did not follow for DBS-ON. Moreover, we found a trend toward significant difference in random switch between both stimulation conditions. A possible explanation for these findings might be a more exploratory and impulsive decision making of PD patients due to STN-DBS.

Our analysis of confidence level showed no effect of DBS on metacognitive assessment. Using signal detection theory type 2, we found no significant difference between d and meta-d for both DBS-ON and DBS-OFF which leads to the conclusion that for both stimulation conditions, patients assess the same information for both type 1 decision-making and type 2 confidence rating reflecting self-awareness. Therefore, a possible impairment in decision making is not based on lack of self-awareness promoted by STN-DBS.

With regards to decision-making and uncertainty-monitoring the medial prefrontal cortex (mPFC) has been reported to play a major role in conflict situation and processing of evidence (Cavanagh, Frank, Klein, & Allen, 2010). In particular, theta-power (4-8 Hz) indexed significant differences associated to a high conflict situation between healthy controls and PD patients under DBS-OFF, which did not follow in the case of DBS-ON^16^. Our data revealed significant differences in power between DBS-OFF and DBS-ON regardless of confidence level, which is consistent with previous studies^32^ and indicates an effect of stimulation. However, we were not able to identify an electrophysiological marker of metacognitive confidence, as no significant difference in power of trials corresponding to low and high confidence level was revealed.

The electrophysiological data obtained in our study was able to capture the effect of stimulation, although did not show any significant relationship with metacognition and reversal learning parameters. Nevertheless, it would be interesting to explore a possible effect of DBS on the mentioned cognitive processes in relation to brain correlates via combined invasive recordings from deep brain areas such as the STN and non-invasive neurophysiological recordings such as EEG^33^. The advantage of this experimental setting would be higher spatial resolution to clarify more directly the role of the STN and its effects in metacognition.

Our study has limitations. First, the considered PD patient cohort is small, which likely prevented us from detecting significant differences in RL and electrophysiological parameters. Note also that testing patients under medication off for the conditions DBS off/on is a challenging task, particularly if patients perform demanding cognitive tasks. Therefore, future multicenter studies should be directed towards increasing patient sample size and statistical power. Second, our cohort of patients is highly inhomogeneous which means that there are differences in character, preferences, demographics, life experiences and intellectual ability, which might have influenced our results.

## Data Availability

Data of this study are available from the corresponding author upon reasonable request.
